# Perspective and agency during video gaming influences spatial presence experience and brain activation patterns

**DOI:** 10.1186/1744-9081-8-34

**Published:** 2012-07-19

**Authors:** Michael Havranek, Nicolas Langer, Marcus Cheetham, Lutz Jäncke

**Affiliations:** 1 Clinic for Affective Disorders, University Clinic of Psychiatry Zurich, Zurich, Switzerland; 2 Department of Neuropsychology, Psychological Institute, University of Zurich, Zurich, Switzerland

**Keywords:** Spatial presence, Brain activation, Perspective, Agency, EEG, LORETA, Fronto-parietal network, Posterior parietal cortex, Premotor cortex

## Abstract

**Background:**

The experience of spatial presence (SP), i.e., the sense of being present in a virtual environment, emerges if an individual perceives himself as 1) if he were actually located (*self-location*) and 2) able to act in the virtual environment (*possible actions*). In this study, two main media factors (*perspective* and *agency*) were investigated while participants played a commercially available video game.

**Methods:**

The differences in SP experience and associated brain activation were compared between the conditions of game play in first person perspective (1PP) and third person perspective (3PP) as well as between agency, i.e., active navigation of the video game character (active), and non-agency, i.e., mere passive observation (passive). SP was assessed using standard questionnaires, and brain activation was measured using electroencephalography (EEG) and sLORETA source localisation (standard low-resolution brain electromagnetic tomography).

**Results:**

Higher SP ratings were obtained in the 1PP compared with the 3PP condition and in the active compared with the passive condition. On a neural level, we observed in the 1PP compared with the 3PP condition significantly less alpha band power in the parietal, the occipital and the limbic cortex. In the active compared with the passive condition, we uncovered significantly more theta band power in frontal brain regions.

**Conclusion:**

We propose that manipulating the factors *perspective* and *agency* influences SP formation by either directly or indirectly modulating the ego-centric visual processing in a fronto-parietal network. The neuroscientific results are discussed in terms of the theoretical concepts of SP.

## Background

When confronted with well-designed virtual reality (VR) scenarios, many people experience a subjective sense of actually being in the VR environment while transiently being unaware of the technology that delivers the stream of virtual input to the senses. This specific feeling has been coined “spatial presence” (SP) [1-3]. This definition of SP emphasises the important role of spatial cues and the subjective strategy involved in recruiting attentional resources for processing sensory input (perception-oriented approach). An alternative view has been proposed by Sanchez-Vivez and Slater [4]. They underline the particular contribution of supported actions in the real or virtual environment (VE) as a constituent feature of the experience of reality. They argue that the sense of “being there” in a VE is strongly grounded on the ability “to do there” (action-oriented approach).

In the context of a more recent theoretical paper, both approaches are combined and extended [5]. In this theoretical account, a psychological construct referred to as “egocentric reference frame” (ERF) is used to explain SP. An ERF is a mental model of the world organised from a first-person perspective and contains information about the spatial properties of the immediate surroundings. A mediated environment (e.g. VR) offers an alternative ERF to the users’ real-world ERF. The sense of SP emerges if this alternative ERF is chosen as the primary ERF over the competing ERF of the real physical world. The outcome of this selection process is considered to be based on two critical questions: whether a person perceives himself 1) as if actually located in the mediated environment (*self-location*) and 2) as being able to act in the mediated environment (*possible actions*).

Only a few studies to date have examined the neural underpinnings of SP. For example, Baumgartner et al. [6,7] identified a distinct network of fronto-parietal brain regions involved in the generation and regulation of SP during exposure to virtual roller coaster scenarios. Using EEG [6], they found activations in parietal brain regions and deactivations in frontal brain regions in participants experiencing high SP compared with participants experiencing low SP. They concluded from their findings that spatial cues presented in the roller coaster scenario generate a sense of SP by strongly activating parietal brain regions (in the dorsal visual stream particularly) that thus lead to enhanced egocentric processing of the displayed environment. In a subsequent fMRI study [7], they concluded that frontal (de)activations relating to differences in SP are closely associated with modulatory processes in the dorsolateral prefrontal cortex (DLPFC). Specifically, they concluded that activation in the DLPFC modulates the sense of SP by down-regulating egocentric visual processing in the dorsal visual stream, as reflected in those participants who experience low SP. This interpretation has received support by further studies using EEG [8] and transcranial direct current stimulation (tDCS) [9], the latter relating these ideas to neuroscientific concepts of behaviour and impulse control [for review, see [10]], and by an interesting study investigating flow experience during video game playing [11].

Considering the increasing importance of VR in our society (e.g. in TV, video gaming and the internet), this study aimed to extend our understanding of SP by investigating the neural correlates of the theoretically proposed key stages in the formation of SP. It was decided to experimentally influence the choice of the virtual ERF as the primary ERF by manipulating the impression of *self-location* on the one hand and the perceived *possible actions* on the other hand. To do so, the two important media factors *perspective* (first-person perspective vs. third-person perspective) and *agency* (being the agent of the actions in the VR vs. being the observer of the actions in the VR) were experimentally controlled and manipulated. These two factors have been shown to influence experience of SP in various psychometrical and behavioural studies [12,13]; however, no study has investigated their influence on SP on a neural level. Thus, the present study sought to establish how manipulating the factors *perspective* and *agency* would influence the impression of *self-location* and the perceived *possible actions* and the formation of SP on a perceptual and a neural level.

To investigate this, electroencephalography (EEG) was recorded and subjective SP experience was assessed while participants played the commercially available video game “The elder scrolls 4: Oblivion”. Participants had to play a simple, non-arousing task, in which they were required to navigate the video game’s avatar (i.e. the virtual character) to a given location in four different conditions (i.e. in a 2x2 design combining the factors *perspective* and *agency*). The two *perspective* conditions entailed playing in the 1PP or in the 3PP. The two *agency* conditions allowed participants to either actively control the avatar (active) or to passively observe the avatar while it was in fact controlled by another player (passive). EEG registration was chosen over fMRI measurements to facilitate presentation of a real-life gaming situation and because EEG has been shown to be a valuable tool in the study of video and computer gaming in previous research [14,15]. In order to directly compare our EEG results with the previously obtained functional magnetic resonance imaging (fMRI) and tDCS findings of our group [7,9,10], the sLORETA software (standard low-resolution brain electromagnetic tomography) was used also in order to calculate the cortical sources [16]. Based on previous studies, it was hypothesized that manipulating the factors *perspective* and *agency* influences SP by either directly or indirectly modulating the ego-centric processing in the dorsal stream. The following predictions were thus formulated for our EEG experiment:

H1: Playing the video game under 1PP compared with 3PP will enhance the experience of SP and reveal stronger activation in parietal areas (especially in the dorsal visual stream).

H2: Actively playing the video game compared with passively observing it will enhance the experience of SP and show stronger deactivation in frontal brain areas (e.g. in the DLPFC).

## Methods

### Participants

Twenty healthy male volunteers took part in this experiment (age =19-32 years; *M* = 23.5 years; *SD* = 3.83). One person had to be excluded from statistical analysis due to missing EEG data. All participants were consistently right-handed and native speakers of German. Handedness was assessed using the Annett-Handedness Questionnaire [17]. To measure health status, a questionnaire regarding neurological and psychiatric health, drug use, hearing and visual deficits was used. None of the participants reported any history of neurological or psychiatric disease, sensory impairments or subjective cognitive impairments. To control for any effects of gaming expertise, the sample was stratified according to the gaming experience of the participants into 10 experienced gamers (>5 hours per week, *M* = 11.70 and *SD* = 8.24) and 10 non-gamers (<1 hour per week, *M* = 0.2 and *SD* = 0.42). The participants were recruited from various sources, such as the university, the social network of the investigators and via internet ads. The local ethics committee approved the study and all participants gave written informed consent.

### Experimental task

**Figure 1:**
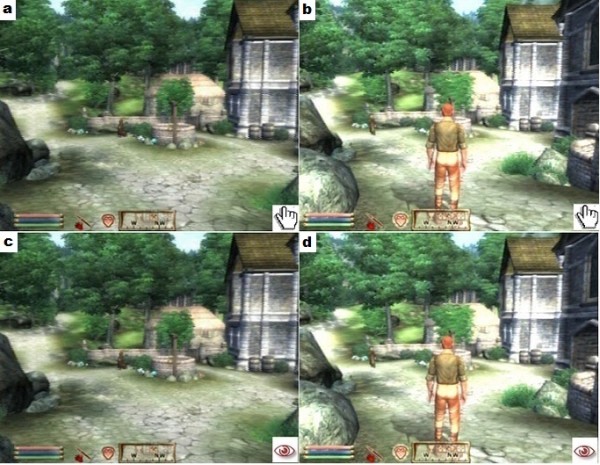
**2x2 factorial design: Playing in the 1PP was compared with playing in the 3PP.** This was further examined in relation to actively playing and controlling the video game avatar or simple passive observation of the avatar controlled by another player. These 4 conditions were: 1PP-active (Figure 1a), 3PP-active (Figure 1b), 1PP-passive (Figure 1c) and 3PP-passive (Figure 1d).

Participants played a specific video game scenario from the commercially available video game “The elder scrolls 4: Oblivion” (Bethesda Softworks LLC, a Zeni Max Media Company). The game was played on the Playstation 3 console (Sony Corporation) using the original Playstation 3 controller, on a TV screen (26 inch) placed at a distance of 1.5 m in front of them. “Oblivion” is a single-player role-playing game in which the player has the ability to explore a richly detailed game world using a realistic looking human avatar (see Figure 1). Because all participants played this game for the first time, a simple scenario was used in which they were required to navigate their avatar to a given location. The selected game scenario was non-arousing, without enemies or other dangerous situations. A 2x2 factorial design with the factors *perspective* and *agency* was used. After a short training session to learn the correct navigation of the avatar, each person performed two simple tasks. In the *active* condition, participants were instructed to complete a mission in which they had to navigate their avatar to follow a specific path and to find the entry of a castle. In the *passive* condition, participants were instructed to closely pursue how the avatar completed the same task but controlled by another player. These tasks were performed by all participants from two different perspectives. In the 1PP, participants performed the task by looking through the eyes of the avatar, that is, as if actually located in its body. In the 3PP, participants performed the task from a viewpoint behind and above the avatar (see Figure 1). Thus, there were 4 conditions (1PP-active, 1PP-passive, 3PP-active and 3PP-passive) each of which was performed once by each participant. Each trial took 2–3 minutes, depending on the speed of each participant in the active conditions. The 4 trials were systematically randomised. During these trials, continuous EEG was recorded. After each trial, participants answered a short questionnaire about their SP experience (see next paragraph).

### Psychometrical measurements

Since the sense of SP is a subjective feeling, the MEC-SPQ questionnaire was used [18]. The MEC-SPQ is specifically designed to measure SP and consists of 9 scales. Four of these cover process factors (attention allocation, spatial situation model, self-location, possible actions), two scales refer to states and actions (higher cognitive involvement, suspension of disbelief) and three scales address more trait-like personality characteristics (i.e. domain specific interest, visual spatial imagery, and absorption). Two of the process scales are specifically intended to reflect physical (or spatial) presence: namely the *self-location* and the *possible actions* scale. The sub-scale *self-location* measures the impression of the subject to be physically in the middle of the virtual game environment (e.g. “I felt like I was actually there in the environment of the presentation.”). The sub-scale *possible actions* assesses the impression of being able to act in the VE (e.g. “The objects in the presentation gave me the feeling that I could do things with them.”). There are three versions of the MEC-SPQ: a full, a medium and a short version of all the scales (with 8, 6 or 4 items per scale). All versions have been validated in large samples in different cities within three countries (Los Angeles, Helsinki, Porto) [18]. In our study, a German translation of the 4-item scale for the sub-scales *self-location* and *possible actions* was used. Participants were instructed to answer on an 8- point scale ranking (“0 = I do not agree at all” to “7 = I agree fully”), instead of the proposed 5-point scale because it was determined in the pilot study that more precise results would be derived from the use of a larger scale. In the statistical analysis, the Friedman test was used as a first step to identify effects of the factors *perspective* and *agency* on the sub-scales *self-location* and *possible actions*. In a second step, the Wilcoxon test was used to make three pairwise comparisons between the four conditions (1PP-active vs. 3PP-active, 3PP-active vs. 1PP-passive and 1PP-passive vs. 3PP-passive). The results were considered significant in case of *p* < 0.05, corrected for multiple comparisons (Bonferroni).

### EEG recordings and preprocessing

EEG was recorded from 30 scalp electrodes using a Brain Vision amplifier system (Brain Products, Germany). Silver-silver-chloride electrodes were used in association with the “Easy Cap System“(International 10–10 system, Falk Minow Services, Germany) and were attached according to the international 10–10 system in the following positions Fp1/2, F3/4, F7/8, Fz, FCz, FT7/8, FC3/4, T7/8, C3/4, Cz, TP7/8, TP9/10, CP3/4, CPz, P7/8, P3/4, Pz, O1/2 and Oz. Recording reference was at FCz, with off-line re-referencing to average reference [19,20]. An electro-oculogram (EOG) was recorded from two additional electrodes placed below the outer canthus of each eye to record horizontal eye movements. Brain Vision Recorder and Analyzer (Brain Products, Germany) were used to record and analyze the data. The digital sampling rate was 500 Hz; impedance was kept below 10kΩ. Participants were seated in a comfortable chair with their head resting in a head mounting within a sound- and electrically shielded chamber with dim illumination. In the beginning of the experiment, a short 3 minute open eye resting condition EEG was taken as baseline recording. Then, after a short training session (to learn the navigation of the avatar), continuous EEG was recorded during each experimental trial.

After recording EEG, the data were bandpass filtered from 1 to 30 Hz using a notch filter at 50 Hz. Then, an independent component analysis (ICA) algorithm provided in the EEGlab software was used to correct for artefacts (e.g. eye blinks & eye movements). It has been shown that ICA can effectively detect, separate and remove activity in EEG records from a wide variety of artificial sources [21]. In addition, all recorded data were carefully and individually checked for artefacts (sweating & muscle artefacts) by visual inspection. The artefact-free EEG was recomputed against the average reference [22]. Each of the four conditions was then segmented into epochs of 2-s duration. We used 68 segments per subject and condition for all the analyses (30.88% of all epochs were excluded).

### Scalp map analysis

A discrete Fourier transformation algorithm was applied to all 2-s epochs according to the frequency band spectra defined by Kubicki et al. [23]: delta (1.5-6 Hz), theta (6.5-8 Hz), alpha1 (8.5-10 Hz), alpha2 (10.5-12 Hz), beta1 (12.5-18 Hz), beta2 (18.5-21 Hz), beta3 (21.5-30 Hz) and omega (1.5-30 Hz). Of these frequency band spectra, the alpha and theta band power interested us the most. Alpha band power has repeatedly been shown to be inversely related to haemodynamic brain responses especially in lateral frontal and parietal brain areas [24-26] and based on these results, we take the alpha band power as an indicator of cortical deactivation in these areas. Theta rhythm in frontal areas is referred to as “frontal midline theta” (FM-theta) and has been observed during a large variety of tasks such as mental calculation, working memory and learning, error processing, and meditation [27,28]. Therefore, FM-theta has been interpreted as a correlate of heightened mental effort and sustained attention required during a multitude of operations. Furthermore, FM-theta has been found in previous EEG based video gaming studies [29-31]. Thus, alpha band power (including alpha1 [8.5-10 Hz] and alpha2 [10.5-12 Hz]) and theta band power (6.5- 8 Hz) spectra were calculated for each single epoch and then averaged across epochs.

In accordance with the study of Baumgartner et al. [6], 12 electrodes were collapsed into three frontal and three parietal electrode clusters: frontal left (F3/FC3), frontal midline (Fz/FCz), frontal right (F4/FC4), parietal left (CP3/P3), parietal midline (CPz/Pz), and parietal right (CP4/P4). In a first step, the two frequency band spectra (alpha and theta) were analysed separately with two four-way ANOVAs (analysis of variance) with the following repeated measurements factors: *perspective* (1PP vs. 3PP), *agency* (active vs. active), *region* (frontal vs. parietal) and *hemisphere* (left, midline and right). Greenhouse-Geisser correction was applied to guard against effects of heteroscedasticity. In a second step, dependent t-tests were used to make four pairwise comparisons between the four conditions separately for alpha and theta (1PP-active vs. 1PP-passive, 3PP-active vs. 3PP-passive, 1PP-active vs. 3PP-active and 1PP-passive vs. 3PP-passive). The statistical analysis was performed with SPSS (version 17). The results were considered significant in case of *p* < 0.05, corrected for multiple comparisons (Bonferroni). In the presentation of the ANOVA results, alpha1 and alpha2 will be reported together as alpha (8.5- 12 Hz).

### sLORETA analysis

The sLORETA software was used for source reconstruction. The sLORETA method [32,33] calculates the three dimensional distribution of electrically active neuronal generators within the brain as current density values (A/cm^2^) based on the recorded scalp electric potential differences. sLORETA estimates the inverse problem using the assumption that the smoothest of all possible activities is the most plausible one. This assumption is supported by electrophysiological data demonstrating that neighbouring neuronal populations show highly correlated activity [34]. The sLORETA software implies a three-shell spherical head model registered to the Talairach and Tournoux atlas (Brain Imaging Centre, Montreal Neurological Institute). Source solutions space was limited to cortical gray matter and hippocampus according to the probability atlas provided by the Montreal Neurological Institute (number of voxels: 6392, voxel dimension: 5 mm^2^). It has been shown in various studies that sLORETA is able to correctly locate with fairly low errors [35-38], which has been validated from studies combining sLORETA with fMRI [39] and with PET [40]. Statistical significance is assessed by means of a nonparametric randomization test [41] and thresholds were set to *p* < 0.05 (corrected for multiple comparisons).

## Results

### Behavioural results

**Figure 2:**
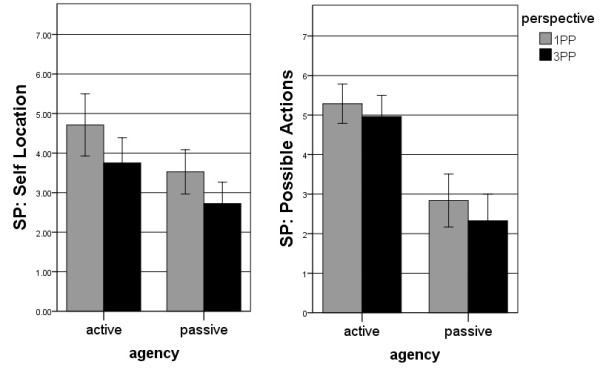
**SP rating.** Depicted are the means (with *SE*) in the *self-location* rating (Figure 2a) and in the *possible actions* rating (Figure 2b) on an 8-point scale. This is broken down for the four different conditions: 1PP-active, 3PP-active, 1PP-passive and 3PP-passive. The subjects indicated the greatest SP experience in 1PP-active, followed by 3PP-active, 1PP-passive and 3PP-passive in this order.

Comparing SP ratings, significant main effects were found in both sub-scales (Friedman test: *self-location*: *χ²* = 25.46, *df* = 3, *p* < 0.001; *possible actions*: *χ²* = 47.50, *df* = 3, *p* < 0.001). Comparing the four conditions pairwise, a significant difference was found between 1PP-active and 3PP-active (*Z* = 2.68, *p* < 0.01) and between 1PP-passive and 3PP-passive (*Z* = 2.60, *p* < 0.01) but not between 3PP-active vs. 1PP-passive (*Z* = 0.50, *p* > 0.05) for *self-location*. Figure 2a illustrates greater SP in 1PP than in 3PP and this more so in the active condition than in the passive. For *possible actions,* the same pattern of SP ratings was observed, namely more SP in 1PP than in 3PP and more SP in the active than in the passive condition (see Figure 2b). But in this condition the comparison between 1PP-active and 3PP-active was not significant (*Z* = 1.57, *p* > 0.05), whereas the comparisons between 3PP-active and 1PP-passive (*Z* = 3.71, *p* < 0.001) and between 1PP-passive vs. 3PP-passive (*Z* = 2.16, *p* < 0.05) were significant. As shown in Figures 2a and 2b, *agency* had a larger absolute influence on both perspective conditions: Higher SP ratings were found for the active conditions (1PP-active and 3PP-active) than for the passive conditions (1PP-passive and 3PP-passive). In all of these analyses, no significant differences were found between gamers and non-gamers.

### Scalp map analysis

**Figure 3:**
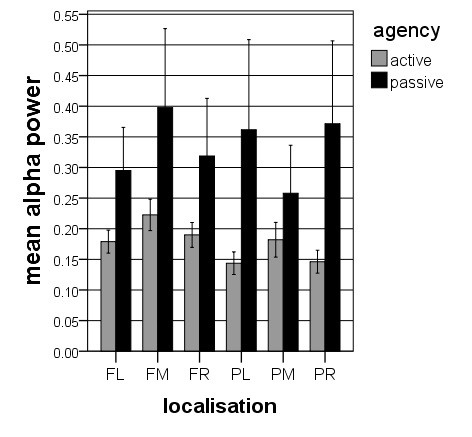
Alpha band power foragency*localization. Illustrated are the means (with *SE*) of the interaction effect for the factor *agency* * *localization*, indicating more alpha band power in passive than in active.

**Figure 4:**
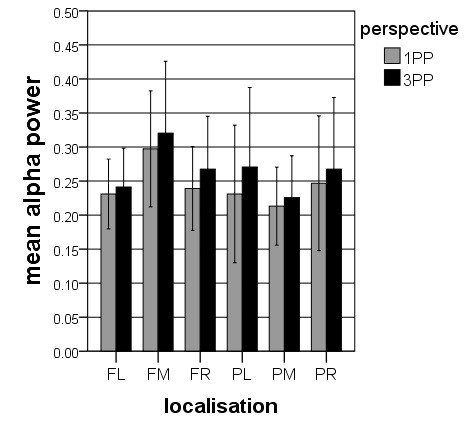
Alpha band power forperspective*localization. Depicted are the means (with *SE*) of the interaction effect for the factor *perspective* * *localization*, indicating more alpha band power in 3PP than in 1PP.

The alpha- and theta power values were subjected to four-way ANOVAs with *perspective* (1PP vs. 3PP), *agency* (active vs. passive), *region* (frontal vs. parietal), and *hemisphere* (left, midline and right) as factors. The ANOVA for the alpha power revealed two significant main effects for *agency* (*F* = 5.306, *df* = 1, 17, *p* < 0.05) and for *perspective* (*F* = 6.841, *df* = 1, 17, *p* < 0.05) and three significant interaction effects for *agency* * *perspective* (*F* = 5.459, *df* = 1, 17, *p* < 0.05), for *agency* * *hemisphere* (*F* = 4.806, *df* = 2, 16, *p* < 0.05), and for *region* * *hemisphere* (*F* = 5.033, *df* = 2, 16, *p* < 0.05). The effect for *agency* is qualified by larger alpha band power during the passive condition compared to the active condition. The effect for *perspective* depends on larger alpha band power during 3PP than during 1PP. The interaction between *agency* * *perspective* is qualified by larger alpha band power during 3PP than during 1PP only in the passive condition but not in the active condition. The interaction effect for *agency* * *hemisphere* demonstrated topographically the strongest alpha band power for the midline electrode clusters for the active condition and a similar distribution between the left, the midline and the right electrode clusters for the passive condition. Finally, the interaction effect for *region* * *hemisphere* revealed in frontal areas more alpha band power in the midline electrode cluster compared with the lateral electrode clusters, and in parietal areas more alpha band power in the lateral electrode clusters compared with the midline clusters. Table 1 shows the results of the post-hoc t-tests between the four conditions. In summary, and as depicted in Figures 3 and 4, there is generally more alpha band activity in the passive compared with the active condition and in the 3PP compared with 1PP.

**Table 1:** post-hoc t-tests

		***M***	***t***	***df***	***p***
**Alpha**	1PP-active vs. 1PP-passive	−0.12	−1.96	18	>0.05
	3PP-active vs. 3PP-passive	−0.17	−2.38	18	<0.05*
	1PP-active vs. 3PP-active	0.00	−1.28	18	>0.05
	1PP-passive vs. 3PP-passive	−0.05	−2.82	18	<0.05*
**Theta**	1PP-active vs. 1PP-passive	0.05	2.80	18	<0.05*
	3PP-active vs. 3PP-passive	0.03	1.85	18	>0.05
	1PP-active vs. 3PP-active	0.00	−0.41	18	>0.05
	1PP-passive vs. 3PP-passive	−0.03	−1.72	18	>0.05

Summarized are the results of the post-hoc t-tests between the four conditions separately for alpha and theta. The results were considered significant in case of *p* < 0.05, corrected for multiple comparisons.

**Figure 5:**
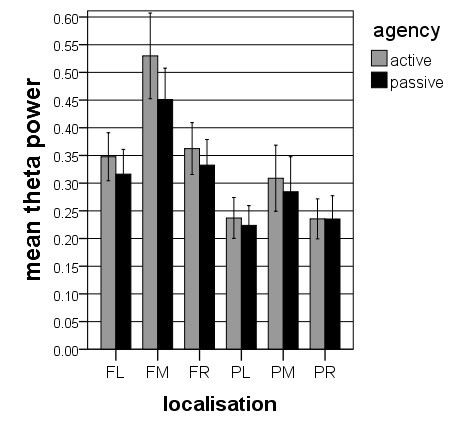
Theta band power for*agency* *localization. Illustrated are the means (with *SE*) of the interaction effect for the factors *agency* * *localization*, indicating more theta band power for active than passive.

**Figure 6:**
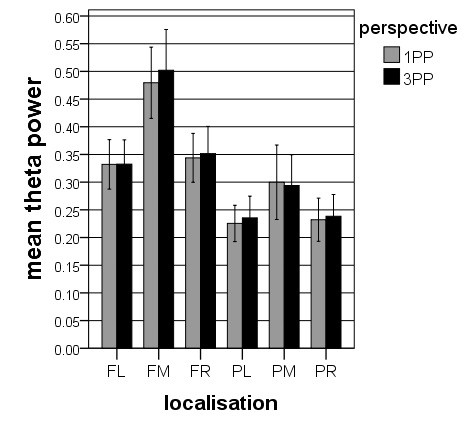
Theta band power forperspective*localization: Depicted are the means (with *SE*) of the interaction effect for the factor *perspective* * *localization*, indicating no significant differences between the perspectives.

The ANOVA for the theta band power showed three significant main effects for *agency* (*F* = 5.731, *df* = 1, 17, *p* < 0.05), for *region* (*F* = 39.265, *df* = 1, 17, *p* < 0.001) and for *hemisphere* (*F* = 38.521, *df* = 2, 16, *p* < 0.001), and three significant interaction effects for *agency* * *region* (*F* = 16.406, *df* = 1, 17, *p* < 0.001), for *agency* * *hemisphere* (*F* = 8.032, *df* = 2, 16, *p* < 0.001) and for *region* * *hemisphere* (*F* = 8.973, *df* = 2, 16, *p* < 0.01). However, there was neither a significant main effect nor a significant interaction effect for the factor *perspective* (main effect: *F* = 1.473, *df* = 1, 17, *p* > 0.05). Unlike in the alpha band ANOVA, there was more theta band power in the active condition than in the passive. In addition, the main effects for *region* and for *hemisphere* and the interaction effect for *region* * *hemisphere* showed significantly more theta band power in frontal brain parts and significantly more theta in the midline electrode clusters. The interaction effect for *agency* * *hemisphere* demonstrated increased theta band power at the midline electrode clusters for the active condition compared to the passive condition. The interaction effect for *agency* * *region* showed more theta band power in the frontal electrode clusters in the active condition compared to the passive. Table 1 shows the results of the post-hoc t-tests between the four conditions. In summary, Figures 5 and 6 illustrate that there are significant main and interaction effects for *agency* but not for *perspective*.

### sLORETA analysis

**Figure 7:**
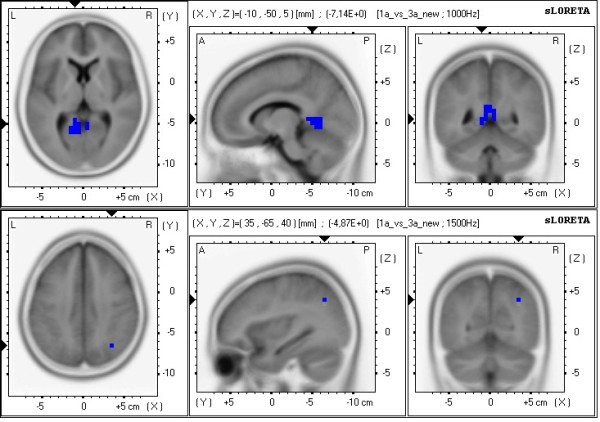
**sLORETA images of the comparison 1PP-active vs. 3PP-active.** Shown are the functional sLORETA images for the comparison 1PP-active vs. 3PP-active in the alpha1 frequency band (Figure 7a) and alpha2 frequency band (Figure 7b). Increased activity in 1PP-active compared with the 3PP-active condition is labeled red, decreased activity is labeled blue.

**Figure 8:**
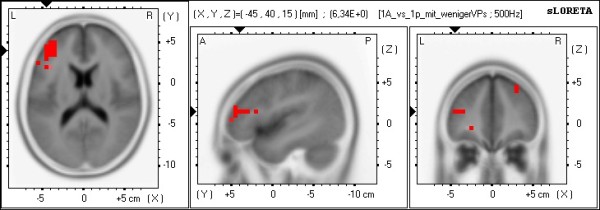
**sLORETA images of the comparison 1PP-active vs. 1PP-passive.** Shown are the functional sLORETA images for the comparison 1PP-active vs. 1PP-passive in the theta frequency band. Increased activity in 1PP-active compared with the 1PP-passive condition is labeled red, decreased activity is labeled blue.

Using sLORETA to locate the neural underpinnings (Table 2 summarizes the sLORETA results), the comparison between 1PP and 3PP (in the active condition) revealed in 1PP significant less alpha1 frequency band power in the limbic cortex (bilateral posterior cingulate, left cingulate cortex, left parahippocampalgyrus) and the occipital cortex (left lingual gyrus, left cuneus) and significant less alpha2 frequency band power in the parietal cortex (right inferior parietal lobule) [see Figures 7a/b]. The comparison between active and passive (in 1PP) revealed in the active condition compared to passive significantly more theta frequency band power in frontal brain parts bilaterally (superior, middle, medial and inferior frontal gyrus in the left hemisphere and superior and middle frontal gyrus in the right hemisphere) [see Figure 8].

**Table 2:** sLORETA results

**X**	**Y**	**Z**	**BA**	**Voxel Value**	**Lobe**	**Main Structure**	**Cluster including**
**1PP-active vs. 1PP-passive (theta)**							
−45	40	15	46	6.34E + 00	Frontal Lobe	Inferior Frontal Gyrus	Superior, middle, medial, inferior frontal gyrus
30	40	40	9	5.42E + 00	Frontal Lobe	Middle Frontal Gyrus	Superior, middle frontal gyrus
**1PP-active vs. 3PP-active (alpha1)**							
−10	−50	5	29	−7.14E + 00	Limbic Lobe	Posterior Cingulate	Posterior cingulate, cingulate gyrus, parahippocampal gyrus, lingual gyrus, cuneus
5	−50	5	29	−5.32E + 00	Limbic Lobe	Posterior Cingulate	Posterior cingulate
**1PP-active vs. 3PP-active (alpha2)**							
35	−65	40	39	−4.87E + 00	Parietal Lobe	Inferior Parietal Lobule	Inferior parietal lobule
**High SP vs. Low SP (alpha2)**							
−25	−60	45	7	−4.06E + 00	Parietal Lobe	Superior Parietal Lobule	Superior parietal lobule, precuneus

Summarized are the sLORETA results of significant differences in brain electrical activity in different comparisons and for different frequency bands. Coordinates are defined in the standard stereotaxic space of Talairach and correspond to the observed activation maxima. The row “Cluster including” describes the extent of the activated brain region. The results were considered significant in case of *p* < 0.05, corrected for multiple comparisons.

**Figure 9:**
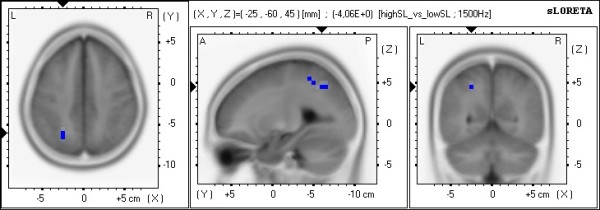
**sLORETA images of High SP vs. Low SP.** Shown are the functional sLORETA images for the comparison High SP vs. Low SP in the alpha2 frequency band. Increased activity in High SP compared with the Low SP condition is labeled red, decreased activity is labeled blue.

To compare the present EEG study with previous EEG and fMRI studies of Baumgartner et al. [6,7], two more analyses were conducted to compare the four conditions. First, the condition that induced the highest SP experience (number of participants with the highest SP in 1PP-active: 14, 3PP-active: 3, 1PP-passive: 3 and 3PP-passive: 0) was compared with the condition that induced the lowest SP (number of participants with the lowest SP in 1PP-active: 1, 3PP-active: 2, 1PP-passive: 2 and 3PP-passive: 15) for each participant. This comparison showed significantly less alpha2 frequency band power in parietal brain regions of the left hemisphere (left superior and inferior parietal gyrus, left precuneus) in the high SP conditions compared with the low SP conditions [see Figure 9.

**Figure 10:**
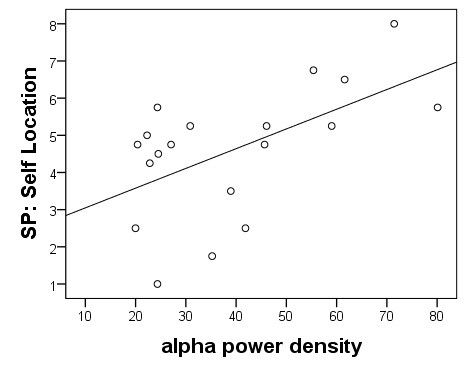
**Correlation between SP ratings and brain activation.** Depicted is the correlation between the SP rating in *self-location* (one dot is the value of one participant) and the mean brain activation (alpha band power in mV) in the DLPFC of both hemispheres (left hemisphere: -30; 15; 15, right hemisphere: 30; 15; 15).

Second, a region of interest analysis for the DLPFC was performed. The mean intracerebral activation in the DLPFC of each hemisphere (left hemisphere: -30; 15; 15, right hemisphere: 30; 15; 15) was estimated and correlated with the SP ratings of the *self-location* subscale. By this, a significant correlation between SP and the deactivation in the DLPFC of both hemispheres was found: High SP ratings were correlated with the intracerebral alpha activity in both DLPFC (left hemisphere: *r*_*s*_ = 0.526, *p* < 0.05; right hemisphere: *r*_*s*_ = 0.566, *p* < 0.05; both hemispheres: *r*_*s*_ = 0.563, *p* < 0.05) [see Figure 10].

## Discussion

The goal of this study was to establish whether SP is modulated by the two critical media factors *perspective* and *agency* and to delineate the neural impact of these factors during video game play. We thus conducted an experiment in which the participants played the video game “Oblivion” in four different conditions (1PP-active, 3PP-active, 1PP-passive and 3PP-passive) while scalp EEG was recorded and subjective experience of SP was assessed. The most important findings may be summarised as follows: (1) Higher SP ratings were obtained in 1PP compared with 3PP and in the active compared with the passive condition. (2) The analysis of the scalp EEG data revealed more alpha band power during 3PP than during 1PP and more alpha band power in the passive than in the active condition. (3) For the theta band, there was more activity in the active than in the passive condition and no significant activity difference between the two perspectives. (4) The intracerebral activations inferred with sLORETA revealed less alpha band activity in the parietal cortex, the occipital cortex, and the limbic cortex for the 1PP compared to the 3PP condition. (5) Finally, there was stronger theta activity bilaterally in the frontal cortex including superior, middle and inferior areas in the active than in the passive condition. In the following, we will discuss these findings in the context of the literature on SP with specific emphasis on neuroscientific findings.

### SP feeling in the context of perspective and agency

Operating the video game in the 1PP and active conditions was associated with the strongest subjective SP feelings, thus corroborating the results of previous studies [12,13]. Nevertheless, our findings go beyond these previous studies because we also examined the interaction of these two factors. Doing this, we found stronger SP ratings during the active conditions (1PP-active and in the 3PP-active) than during both passive conditions (1PP-passive and 3PP-passive), meaning that this general effect was independent of whether the subjects played in 1PP or 3PP. Thus, the factor *agency* exerts a stronger perception of SP than the factor *perspective*.

Early conceptualisations of SP have highlighted the specific role of perception on the generation of SP (the perception-oriented view). In this view, SP most strongly depends on the specific perception of spatial cues in VR. It has been argued that the person is concurrently “inattentive” to the spatial cues of the real physical surroundings [2]. Thus, a sensory-rich media stimulation evokes greater attentional processing preferentially via the visual dorsal stream and thus contributes to an enhanced perception of SP [1,10,18]. An alternative view of SP has been proposed by Sanchez-Vives and Slater (the action-oriented view) [4]. These authors highlight the role of controlled actions in the real or VE as a constituent feature for experiencing SP. The sense of “being there” in a VE is thought to be based on the ability to “do there”. Thus, conscious action control and movement within the VE is an important constituent of SP. This does not necessarily mean that real actions must be executed in a VE. A mental representation of an action can be automatically triggered by the incoming VE stimuli irrespective of subsequent execution of the action or not [10]. Both views, the perception-oriented and the action-oriented, receive support from our own study since we demonstrate that the experience of SP can be evoked by the perceptual *perspective* and by the experience of *agency* in the VE.

However, our experiment indicates that *agency* is more important for SP, given the larger influence of *agency* than of *perspective* on SP ratings. This finding is consistent with the action-oriented view in a gaming situation, although this may be limited to the specific characteristics of computer and video gaming and may not be generalized to all VR situations. In a common video gaming situation like the one used in our experiment the sensory modalities are not exposed to the kind of near-real-life stimuli that more sophisticated VR technologies can deliver. Sophisticated VR technologies are capable of addressing different sensory modalities and of sustaining a match between these different modalities (e.g. by using eye goggles and real-time tracking devices) [42]. However, in video or computer gaming, the means to stimulate sensory modalities are technically limited to a TV or computer screen. But we suggest that in video gaming this limitation can be compensated for by an increase in the interactivity enabled or required by the mediated environment. Referring to the model of Wirth et al. [5], we postulate that the possibility to act and manipulate first “attracts” the attention of the player to the VE and, in a second step, increases the acceptance of the mediated ERF as the primary ERF. Consequently, it is possible that (especially in video gaming) the perception of *agency* is of higher importance than the perception of *self-location*.

### Neurophysiology in the context of perspective and agency

With respect to the cortical activation pattern, we identified decreased alpha band activity in the active and 1PP conditions. Since the active and 1PP conditions evoke the strongest SP perception, it is clear that strong SP is associated with less alpha band activity. Based on studies that revealed strong negative relationships between hemodynamic responses and alpha band power [24-26], we take the alpha band power as an indicator of cortical deactivation in these areas. Thus, higher cortical activation (strong hemodynamic responses) is associated with less alpha band activity. In the context of our study, the weaker alpha band responses over frontal and over parietal areas during 1PP and the active conditions indicates increased brain activation during these conditions. Since these conditions are also those with the strongest sense of SP, we assume that strong SP is associated with increased activation in the fronto-parietal network. For the theta band, we identified more theta band power (especially over the frontal midline positions) during the active conditions. In contrast to alpha band activity, theta band activity is not a direct indicator of neural activation or deactivation. However, theta band activity, especially over the frontal midline electrodes (FM-theta), is known to be involved in the control of complex cognitive operations such as working memory, memory processes, and the control of actions [27,28]. Furthermore, our finding of increased FM-theta in active conditions is consistent with previous EEG based video gaming studies in showing that operating actions in VEs is associated with increased FM-theta [29-31].

### Intracerebral activations in the context of SP feeling

In the comparison between high SP conditions and low SP conditions, we found less alpha2 frequency band power in parietal brain regions of the left hemisphere (left superior and inferior parietal gyrus, left precuneus) in the high SP conditions. Additionally, we revealed a significant positive correlation between intracerebral alpha activity in the bilateral DLPFC and SP ratings of the self-location subscale. These findings are in accordance with previous findings of our group in which we demonstrated 1) that SP experience is associated with the involvement of a distributed fronto-parietal network including the dorsal visual stream as a prominent part, and 2) that the DLPFC is one of the major regulators within this network [6,7]. Nevertheless, our results not only replicate previous findings but expand current knowledge by showing that these neurophysiological mechanisms are not confined to the virtual roller coaster scenarios used by Baumgartner et al. [6,7]. Since the roller coaster scenarios were non-interactive and arousing, while our experimental scenarios were interactive and non-arousing, we demonstrate that the core regions associated with SP are relevant in different virtual environments.

### Intracerebral activations in the context of perspective and agency

Comparison of the intracerebral sources of alpha activity between 1PP and 3PP (separately for the active and the passive conditions) revealed a network of neural sources including the parietal cortex (right inferior parietal lobule), the occipital cortex (left lingual gyrus, left cuneus), and the limbic cortex (bilateral posterior cingulate, left cingulate cortex, left parahippocampalgyrus) with decreased alpha activity. Comparison of the active and passive conditions showed stronger theta activity in frontal brain areas during the active conditions (superior, middle, medial and inferior frontal gyrus in the left hemisphere, and superior and middle frontal gyrus in the right hemisphere). The fronto-parietal network found in the present study (and in previous studies of our group) is not exclusively associated with generating and controlling the experience of SP [see also [10]. These regions of the parietal cortex are known to be involved in spatial processing, mental rotation, and most importantly, a part of this network constitutes the dorsal visual stream, which is known to be involved in the egocentric processing [43,44]. These parts of the limbic cortex are known to play an important role in episodic memory, emotional stimuli processing, and action control [23,35]. The identified frontal brain areas are known to be involved in various executive functions that exert regulatory control over emotions and actions with the DLPFC being an important part [27,45-51].

Among the various brain regions within this fronto-parietal network, the role of a parietal-premotor connection associated with sensory-motor integration is particularly interesting for the interpretation of our findings for *perspective* and *agency*. The posterior parietal cortex (PPC) is known to integrate information from different sensory modalities to form a coherent multimodal representation of space coded in a body-centered reference frame. This integration of multisensory cues includes mapping the position of objects in reference to one’s own body [52]. The premotor cortex, (PMC) on the other hand, appears to define a corresponding motor space consisting of all potential motor actions within the surrounding space [53]. It has been suggested that potential target objects within the visual field elicit motor schemas for potential actions that map the position of these objects in the surrounding environment [54]. Put simply, the PPC directly maps the position of objects in reference to the body, whereas the PMC creates a motor space as an intermediate step first.

According to the model of Wirth et al. [5], an alternative egocentric reference frame (ERF) to the real-world ERF develops from spatial cues and action cues of the VE. This ERF contains information about the spatial properties of immediate surroundings from a first-person perspective. The parietal-premotor connection might play a key role in this process. As mentioned in a previous review [10], we believe that the new egocentric view derived from VR is generated in the context of several transformation processes in the dorsal visual stream of the parietal cortex [see [43,44]]. Regarding our findings for *perspective* and *agency*, we propose that mediated by the PPC, spatial cues from the VE directly define the spatial properties within an egocentric view. On the other hand, mediated by the PMC, actions cues in the VE are used to define a motor space which indirectly facilitates an egocentric view. Thus, we propose that both parietal and frontal regions are involved in the generation of the alternative ERF derived from the mediated environment.

The sense of SP is thought to emerge when the ERF from the mediated environment is selected as the primary ERF over the competing ERF of the actual environment (Wirth et al. [5]). We believe that whether or not a person chooses this new ERF as the primary ERF depends (among other things) upon the strength of the influence that media factors such as *perspective* and *agency* exert on this fronto-parietal network. More specifically, we propose that playing in 1PP, as well as actively controlling the actions of the video game avatar, increases the acceptance of the new ERF as the primary ERF mediated by (and observable as) increased activation in the fronto-parietal network.

#### Limitations

The present study has some limitations which should be addressed in future studies. The SP model of Wirth et al. [5], which lays the theoretical foundation for the interpretation of our neuroscientific findings, has not yet been thoroughly investigated empirically. It remains to be seen if this model will receive further support in future investigations.

The post-hoc questionnaires used in this study have disadvantages due to their administration after exposure; these disadvantages include recency effects, anchoring effects and inaccurate recall [55]. However, the alternative of a continuous on-line measurement of SP also has important disadvantages such as disruption of the SP experience itself by drawing attention away from the VE [2]. Future studies may be able to use new developed measurement procedures which seek to overcome these issues.

A limitation of our study design was the fact that we were not able to investigate the factors *perspective* and *agency* independently. In future studies, it may prove beneficial to manipulate the amount of sensory information and the possibilities to act in the VE independently in order to further elaborate how each influence SP and which influences SP more significantly.

## Conclusion

In sum, we propose that manipulating the factors *perspective* and *agency* influences SP by either directly or indirectly modulating the ego-centric visual processing in a fronto-parietal network. The findings of this study fit well within recent neuroimaging and behaviour studies. Several studies have demonstrated that a strong sense of SP is associated with activation in a distributed brain network that specifically includes fronto-parietal regions [6-10]. But our findings are also consistent with different theoretical views on presence (i.e. action- and perception-oriented) and may thus help to reconcile these views in one conceptual framework [5,10].

## Competing interests

The authors declare that they have no competing interests.

## Authors' contributions

MH conducted the literature review, planned the experimental design, performed data collection and data analysis, and prepared all drafts of the manuscript. NL contributed to data analysis and to draft revision. MC contributed to planning the experimental design, contributed to data collection and to draft revision. LJ contributed to draft revision and to theoretical interpretation. All authors have read and approved the final manuscript.
